# A Year Into the Pandemic: The Diversity of Experience Amongst People With Severe Mental Ill Health

**DOI:** 10.3389/fpsyt.2021.794585

**Published:** 2022-01-27

**Authors:** Emily Peckham, Panagiotis Spanakis, Paul Heron, Suzanne Crosland, Gordon Johnston, Elizabeth Newbronner, Ruth Wadman, Lauren Walker, Simon Gilbody

**Affiliations:** ^1^Mental Health and Addiction Research Group, Department of Health Sciences, University of York, York, United Kingdom; ^2^Independent Researcher, Clackmannan, United Kingdom

**Keywords:** schizophrenia, bipolar disorder, severe mental ill health, COVID-19, health risk behavior, inequalities

## Abstract

**Background:**

The COVID-19 pandemic has amplified pre-existing health inequalities and people with severe mental ill health (SMI) are one of the groups at greatest risk. In this study, we explored the effects of the pandemic and pandemic restrictions on people with SMI during the first year of the pandemic.

**Methods:**

We conducted a longitudinal study in a sample of people with SMI. The inception survey was carried out between July and December 2020. Participants were then re-surveyed between January and March 2021. People were contacted by telephone and invited to take part in the study over the phone, online or by postal questionnaire. Across both waves we asked participants about their physical and mental health, health risk behaviors, well-being, loneliness, and employment status.

**Results:**

Three hundred and sixty-seven people with SMI completed the inception survey and 249 people completed the follow up. Whilst some people reported no change in their physical (77, 31%) or mental health (60, 24%) over the course of the pandemic 53 (21%) reported a continuing decline in physical health and 52 (21%) reported a continuing decline in mental health. Participants who maintained a daily routine or reported no decline in physical health were found to be associated with no deterioration in mental health (Daily routine OR 2.27, 95% CI 1.11–4.64; no reported physical health decline OR 0.54, 95% CI 0.17–0.70). Participants were less likely to be occupationally active in the first phase of the pandemic compared to before the pandemic and in the second phase of the pandemic. However, there was no one single experience of people with SMI and similar to studies in the general populations a range of different scenarios was experienced.

**Conclusions:**

We observed a series of factors that might amplify pre-existing health inequalities. Health systems should be mindful of this, and should redouble efforts to set in place changes to practice and policy, which can mitigate these inequalities. Examples might include; raising awareness of the importance of ensuring that people with SMI receive an annual physical health check and supporting people to maintain a daily routine.

## Introduction

People with severe mental ill health (SMI) experience some of the most profound health inequalities of any sector of society, currently experiencing a mortality gap of 15–20 years and instead of decreasing, in recent years, this gap has been increasing (1, 2). Evidence shows that the global COVID-19 pandemic has led to an increase in inequalities, with the most vulnerable sectors of society experiencing both worse outcomes from COVID-19 infection (3) and from the restrictions imposed to reduce the spread of the virus (4). People with SMI are one such group.

Those with poor physical health and poor nutritional status, along with those who have long-term conditions such as diabetes and cardiovascular disease are more at risk of multiple long term conditions (5). Compared with the general population People with SMI have a 78% increase in risk in cardiovascular disease (6) and 12% of people with SMI have diabetes (7) making them one of the groups more at risk from COVID-19. Furthermore, people with chronic conditions are also more at risk from the pandemic restrictions. People with long-term conditions have to manage their condition on a daily basis and require regular health related follow ups, all of which may be affected by the pandemic restrictions. For example, people may experience a lack of or reduced access to health care practitioners and facilities leading to poorer management of their condition which in turn may lead to a worsening of the condition (8). It is not solely people with physical health conditions that are at increased risk; people with existing mental health conditions are also more at risk due to the pandemic restrictions leading to increased social isolation and loneliness (9). Reduced access to services may lead to reduction in routine mental health appointments potentially resulting in a deterioration in their mental health (10).

The optimizing well-being in self-isolation study (OWLS) was set up to explore the effects of the pandemic and the pandemic restrictions on people with SMI. The study comprises of two surveys and two sets of qualitative interviews. The study methods, procedures, and questionnaires were developed in conjunction with our lived experience panel. In this article we compare participants' responses to the initial OWLS survey, conducted between July and December 2020, with their responses to the second OWLs survey, conducted between January and March 2021. The aim being to determine whether there was any change in people's self-reported physical and mental health, well-being, loneliness, and employment status over the course of the pandemic.

## Methods

### Design and Procedures

A sample of people who had previously taken part in The Closing the Gap (CtG) study were invited to take part in the OWLS study. The CtG study was a large clinical cohort (*N* = 9,914) comprising adults (aged 18 years or older) with documented diagnosis of schizophrenia or delusional/psychotic illness (ICD 10 F20.X and F22.X or DSM equivalent) or bipolar disorder (ICD F31.X or DSM equivalent). Ethical approval for the CtG study was granted by West Midlands—Edgbaston Research Ethics Committee (REF 15/WM/0444).

The OWLS study, which is a longitudinal study, recruited a sub-cohort from CTG, to explore the effects of the COVID-19 pandemic restrictions on people with SMI. To be eligible for invitation to OWLS, CtG participants had to have provided contact details and consented to be contacted again, as well as having been originally recruited from a clinical site that had the capacity to collaborate with the University of York research team in a new research project. Eligible participants were then organized in groups based on age, gender, ethnicity, and care setting (primary or secondary mental health care) to ensure representation across many sociodemographic groups. From each group, researchers selected a purposive sample of participants that had most recently participated in the CtG study (e.g., recruited in the last 2 years) ensuring that a range of localities was covered. Recent participation to the CtG was considered important to increase response rates (e.g., the team having current and valid contact details, and participants being familiar with the research team). Locality was used to provide geographical diversity, inviting participants from 17 mental health trusts and six Clinical Research Network (CRN) areas in England, including a mix of rural and urban settings.

Those selected to be invited were contacted by telephone or letter and invited to take part in the OWLS study. Those who agreed to take part were provided with a range of options; (i) to carry out the survey over the phone with a researcher, (ii) to be sent a link to complete the survey online, or (iii) to be sent a hard copy of the questionnaire in the post to complete and return. The full methods of recruitment to the OWLS study have been previously described (11) and are also outlined in the Supplementary Material.

Those who took part in the initial OWLS survey (OWLS 1, T1) were asked if they were willing to complete follow up surveys. We attempted to contact all those who consented to follow up surveys to take part in the follow up survey (OWLS 2, T2). The mean length of time between T1 and T2 was 123 days (SD 40 days). The OWLS study therefore comprised a longitudinal study conducted across the first year of the pandemic.

Ethical approval was granted by the Health Research Authority North West—Liverpool Central Research Ethics Committee (REC reference 20/NW/0276).

### Measures

All variables and analysis reported here have been pre-registered in Open Science Framework (OSF) (https://osf.io/4qwr7/). Links to the OWLS questionnaires can be found in the Supplementary Material. Both OWLS questionnaires were developed in conjunction with our lived experience panel who provided suggestions about items to include and piloted the questionnaires.

#### Self-Reported Changes in Physical and Mental Health

In both OWLS 1 and OWLS 2 participants were asked about changes in their physical and mental health. In OWLS 1 participants were asked the following question about both their physical and mental health “compared to your life before the pandemic restrictions, how would you rate your health in general,” “with the following response options;” “better than before,” “about the same,” “worse than before,” “not sure/don't know.” In OWLS 2 participants were asked “compared to your life 6 months ago, how would you rate your health in general.” With the same response options as in OWLS 1. People who responded “not sure/don't know” were not included in the analysis.

In OWLS 2 participants were asked about their ability to maintain a daily routine with the response options “more than usual,” “about the same,” “less than usual.”

#### Global Well-Being

In both OWLS 1 and OWLS 2 participants were asked the four Office of National Statistics well-being questions (12). (1) overall how satisfied are you with your life, (2) overall to what extent do you feel that the things you do in your life are worthwhile, (3) overall how happy did you feel yesterday, and (4) overall how anxious did you feel yesterday. Response options are scored on a Likert scale of 0–10. For the first three questions a score of 0 indicated not at all and a score of 10 completely and for the final question a score of 0 indicated not at all anxious and 10 indicated completely anxious. After reversing the scores for the anxiety question the scores for the four questions were totaled to give a total well-being score (0–40), with higher scores indicating better well-being.

#### Loneliness

Loneliness was measured at T1 and T2 using the University of California, Los Angeles Loneliness Scale (UCLA-LS) three-item (13) which asks about loneliness symptoms experienced within the past 2 weeks and produces a score range of 3–9, where a higher score indicates greater loneliness.

In addition in OWLS 1 participants were asked whether or not they had been advised to shield (yes/no).

#### Sociodemographic and Health Variables

Sociodemographic variables collected were age, gender (female, male, or transgender), and ethnicity (grouped as White background or Other than White). Age was collapsed into the following bands; 18–30, 31–45, 46–65, and 66 and over. At T1 participants were asked if they were currently receiving support from mental health services (yes/no). To determine employment status at T1 participants were asked about their employment status prior to the pandemic and if their employment status had changed since the pandemic restrictions began. At T2 participants were asked about; their current employment status, whether their financial status had changed (better off, worse off, about the same, don't know, don't wish to answer), and those that were employed were asked if their employment had changed in any way in the last 6 months (reduced or increased hours, reduced or increased pay, furloughed, change in responsibilities). At each time point participants were grouped as being occupationally active if they were in any form of employment (paid, unpaid, or voluntary and hadn't been furloughed) or were a student. We also derived participants' socioeconomic deprivation index according to their postcode (ref from loneliness paper). Index scores range from 1 to 10 with higher scores indicating less deprivation. Scores were grouped as very high deprivation (1, 2), high deprivation (3, 4), medium deprivation (5, 6), low deprivation (7, 8), and very low deprivation (9, 10).

We collected mental health diagnosis details for those who consented to their health records being checked to collect their mental health diagnosis. Diagnoses were categorized into psychosis spectrum disorders (including schizophrenia, schizoaffective, or any other psychotic disorder), bipolar disorder, or other SMI (including participants who were eligible for the CtG study on the basis of a psychosis or bipolar disorder diagnosis which was later changed in their health records e.g., severe depressive disorder with psychotic features). For those who did not consent to access their records or did not provide sufficient identifiable information (e.g., name and date of birth), diagnosis was categorized as “not recorded.” The “not recorded” category was not reported in the pre-registered analysis plan but was added to allow the participants in this category to be retained in the analysis.

#### Digital Communication

Participants were asked at T2 whether they could or could not complete various digital communication tasks and from this a count variable (between 0 and 7) of total tasks participants were able to complete was calculated.

### Statistical Analysis

The study analysis plan was registered on Open Science Framework (https://osf.io/cmgw7). Analyses were undertaken using SPSS v.26. Descriptive statistics were used to describe sociodemographic characteristics, diagnosis details, shielding status, well-being, changes in physical and mental health, loneliness, whether the person was currently receiving support from mental health services and ability to maintain a daily routine.

To test for response bias, we explored whether participants who completed questionnaires at both T1 and T2 differed in age, gender, ethnicity, socioeconomic deprivation, care setting, and diagnosis to those who only took part at T1. Differences were examined with a χ^2^-test (or the likelihood ratio if test assumptions were violated), apart for age where an independent samples *t*-test was used.

Using descriptive statistics we explored the different trajectories of change in mental and physical health across all time points [T1: change compared to before the pandemic, T2: change compared to earlier phase of the pandemic (6 months ago)]. We combined the responses from T1 and T2, generating the following outputs: continuous improvement (better—better), initial improvement followed by stabilization (better—about the same), initial improvement followed by deterioration (better—worse), stable condition (about the same—about the same), initially stable but then improved (about the same—better), initially stable but then deteriorated (about the same—worse), continuous deterioration (worse—worse), initial deterioration followed by improvement (worse—better), initial deterioration followed by stabilization (worse—about the same).

Current Employment status and changes in employment status were described using descriptive statistics. Professional inactivity status at T1 was cross-tabulated with professional inactivity status at T2 and statistically significant differences between the two time points were assessed with the McNemar test.

Using a binary logistic regression model we explored whether a deterioration in mental health during the pandemic restrictions in place between January 2021 and March 2021 (T2) was associated with deterioration in the ability to maintain a daily routine (measured at T2), being advised to self-isolate (measured at T1), loneliness (measured at T2), being professionally inactive (measured at T2), and deterioration in physical health (measured at T2) after adjusting for sociodemographic characteristics, diagnosis, and being seen in secondary care. Each independent variable (the ability to maintain a daily routine, being advised to self-isolate, loneliness, being professionally inactive, and deterioration in physical health) were first individually tested in a univariate regression. Variables with a *p*-value <0.2 were included in the multivariate regression (14). The multivariate regression was hierarchical with all the key sample characteristics (socio-demographic characteristics, diagnosis, and care setting) being in the first block and all other predictors in the second block.

Change in loneliness was examined using a mixed design ANCOVA, with time-point (T1 or T2) as the within subjects factor, age, diagnosis, living alone, and professional inactivity as between subjects factors and digital communication skills as a covariate. Aside from main effects, the two-way interactions between time point and each between-subjects factor were also examined.

## Results

Between July and December 2020, 367 people were recruited to the OWLS study, 315 consented to re-contact, and 249 (79.0%) of those completed a second questionnaire between January and March 2021. Those who did not complete the second survey did not differ from those who did complete the survey, in terms of any of the sociodemographic characteristics, [Age: *t*_(365)_ = −0.45, *p* = 0.650; Gender: Likelihood Ratio_(2)_ = 4.77, *p* = 0.092; Ethnicity: $\chi_{\left(1\right)}^{2}$ = 1.44, *p* = 0.230; Deprivation: $\chi_{\left(4\right)}^{2}$ = 6.47, *p* = 0.167; Care setting: $\chi_{\left(1\right)}^{2}$ = 0.63, *p* = 0.429; Diagnosis: $\chi_{\left(3\right)}^{2}$ = 6.07, *p* = 0.108].

Table 1 gives details of the participant's characteristics. Participants had a mean age of 51.7 years old (range: 21–84), 51.4% were men, 15.6% were from other than White ethnic backgrounds, and 44.6% lived in areas of high/very high deprivation. The primary diagnosis was psychosis-spectrum disorder (48.2%).

**Table 1:** Participant characteristics.

**Characteristic**	***N* (%)** **Total *N* = 249**
**Age**	
18–30	28 (11.2)
31–45	64 (25.7)
46–65	100 (40.2)
66+	57 (22.9)
**Gender**	
Female	116 (46.6)
Male	128 (51.4)
Transgender	5 (2.0)
**Ethnicity**	
Asian	14 (5.6)
Black	4 (1.6)
Mixed	11 (4.4)
White British	200 (80.3)
White (other)	10 (4.0)
Other	10 (4.0)
**Socioeconomic deprivation**	
Very high	60 (24.1)
High	51 (20.5)
Medium	49 (19.7)
Low	43 (17.3)
Very low	38 (15.3)
Missing	8 (3.2)
**Diagnosis**	
Bipolar disorder	83 (33.3)
Psychosis spectrum disorder	120 (48.2)
Other SMI	16 (6.4)
Not recorded	30 (12.0)
**Care setting**	
Primary care	95 (38.2)
Secondary care	152 (61.0)
Missing	2 (0.8)
Employment status^*^	
Employed full time	26 (10.4)
Self-employed	24 (9.6)
Retired	64 (25.7)
Looking after family/home	28 (11.2)
Student	11 (4.4)
Voluntary worker	28 (11.2)
Not employed but seeking work	8 (3.2)
Not employed but not seeking work due to ill health	104 (41.8)
Not employed but not seeking work for some other reason	8 (3.2)
Other	9 (3.6)
**Financial status compared to 6 months ago**	
Better off	51 (20.5)
Worse off	52 (20.9)
About the same	135 (54.2)
Did not wish to answer	2 (0.8)
Missing	9 (3.6)
**Ability to maintain a daily routine**	
No	91 (36.5)
Yes	154 (61.8)
Missing	4 (1.6)
**Shielding**	
Yes	51 (20.5)
No	195 (78.3)
Missing	3 (1.2)
**Current support from mental health services**	
Yes	152 (61.0)
No	95 (38.2)
Missing	2 (0.8)

* *Participants could tick all that applied i.e., someone could be retired and also a voluntary worker*.

Table 2 shows the self-reported changes in mental and physical health, loneliness, well-being, and employment status since the beginning of the pandemic (T1) and the self-reported changes in the mental and physical health, loneliness, well-being, and employment status in the last 6 months (T2, between the first and second phase of the pandemic).

**Table 2:** Self-reported health, professional activity, and changes in employment status.

	**Time point 1 *N* (%)**	**Time point 2 *N* (%)**
**Self-reported global mental health**		
Deterioration	108 (43.4)	85 (34.1)
No deterioration	135 (54.2)	157 (63.1)
**Self-reported global physical health**		
Deterioration	82 (32.9)	95 (38.2)
No deterioration	160 (64.3)	150 (60.2)
		**Mean (SD)**
**Loneliness**	5.85 (2.16)	5.85 (2.19)
**Well-being**	22.73 (8.67)	22.17 (8.68)
**Professional activity**	***N*** **(%)**	***N*** **(%)**
Professionally active	66 (26.5)	88 (35.3)
Professionally inactive	180 (72.3)	160 (64.3)
**Change in employment**		
Reduction in hours	17 (4.6)	13 (5.2)
Reduction in salary	5 (1.4)	13 (5.2)
Change in duties or responsibilities	16 (4.4)	20 (8.0)
Increased hours	8 (2.2)	13 (5.2)
Increased salary	5 (1.4)	8 (3.2)
Furloughed or paid leave	15 (4.1)	12 (4.8)

### Change in Physical and Mental Health

**Figure 1:**
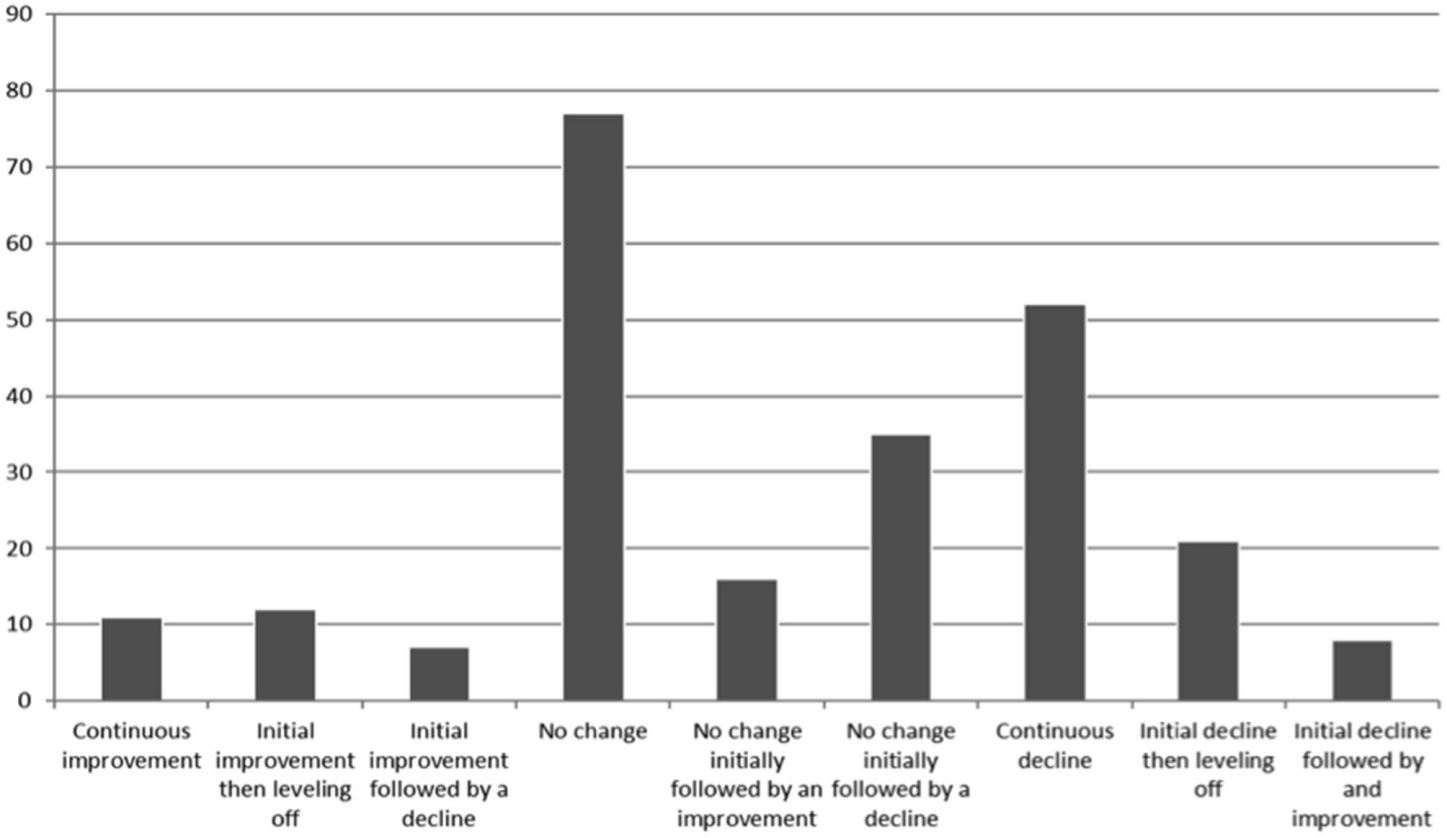
Trajectory of change in physical health.

**Figure 2:**
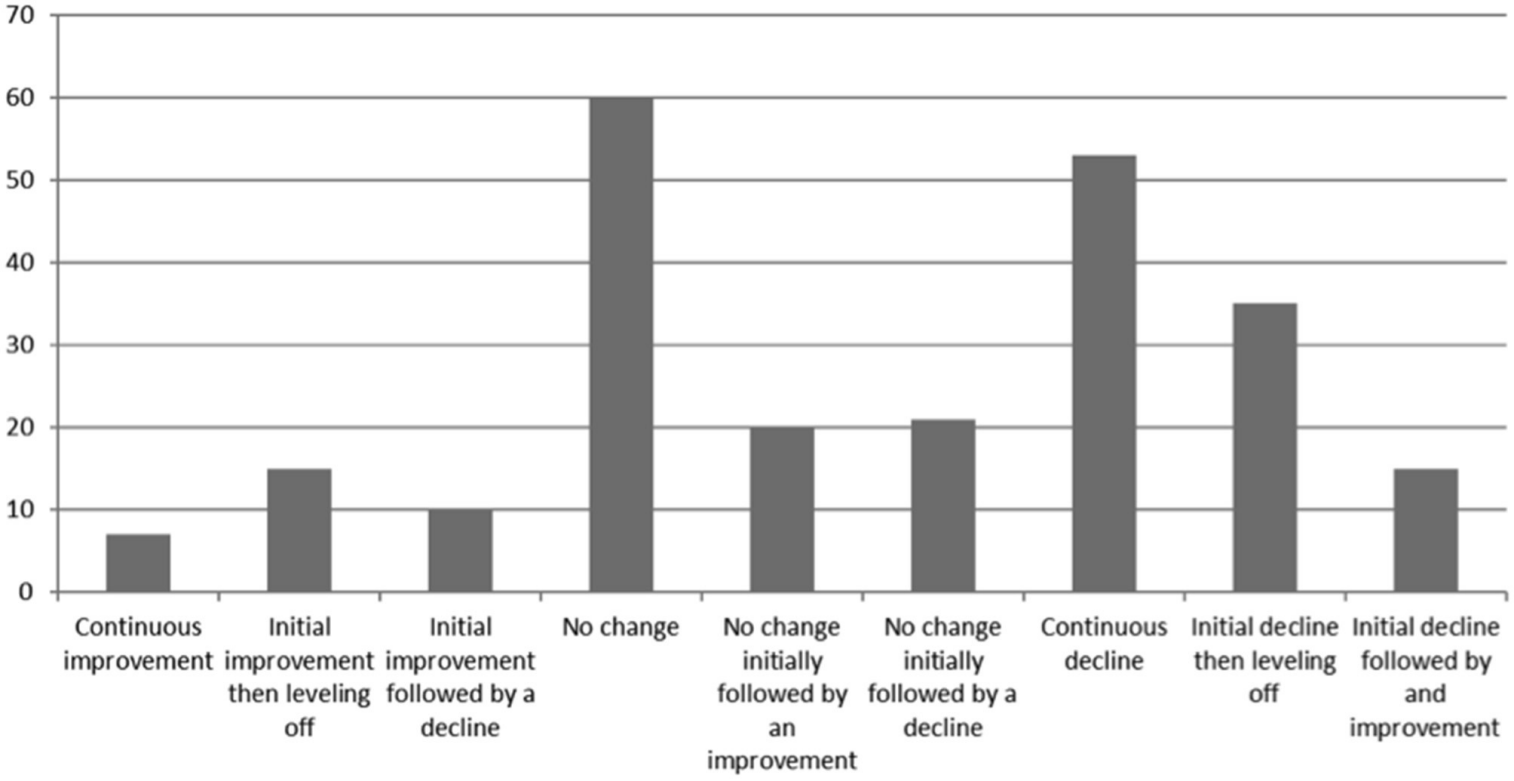
Trajectory of change in mental health.

Figure 1 shows the different trajectories of change in physical health across all time points and Figure 2 shows the different trajectories of change in mental health across all time points.

### Change in Professional Activity

In terms of professional activity 12.1% who were professionally active at T1 became inactive at T2 (Table 3). However, 16.8% of those who were professionally inactive at T1 became professionally active at T2 and this difference was significant (*p* = 0.0). A *post-hoc* test that was conducted to compare levels of professional activity pre-COVID-19 and at T1 (Supplementary Table 1) found that the number of people that were professionally active pre-COVID significantly decreased at T1 (*p* = 0.0). This indicates that while levels of professional activity dropped at T1 they then subsequently increased at T2.

**Table 3:** Professional activity at T1 and T2.

	**Professional activity T1**	
**Professional activity T2**	**Active**	**Inactive**
Active	58 (87.9%)	30 (16.8%)
Inactive	8 (12.1%)	149 (83.2%)

### Change in Mental Health

Ability to maintain a daily routine, loneliness and change in physical health had a *p*-value of <0.2 so were included in the final model. The final model explored whether a deterioration in mental health in the last 6 months was associated with ability to maintain a daily routine, loneliness, change in physical health after adjusting for age, gender, ethnicity, IMDD, diagnosis, and care setting (Table 4). The ability to maintain a daily routine and no decline in physical health were found to be associated with no deterioration in mental health (adjusted OR for change in physical health 0.54, 95% CI 0.17–0.70; adjusted OR for ability to maintain a daily routine 2.27, 95% CI 1.11–4.64).

**Table 4:** Factors associated with a deterioration in mental health.

	**Univariate model**		**Multivariate model**	
	**Odds ratio (95% CI)**	***P***	**Odds ratio (95% CI)**	***P***
**Age**	0.99 (0.97–1.01)	0.17	0.98 (0.97–1.01)	0.12
**Gender**				
Male	1.04 (0.61–1.78)	0.88	1.67 (0.81–3.44)	0.17
Female	1		1	
**Minority status**				
Non-BAME	1.70 (0.78–3.68)	0.18	1.76 (0.68–4.59)	0.25
BAME	1		1	
**IMDD**				
Very high deprivation	0.96 (0.41–2.25)	0.92	0.65 (0.22–1.94)	0.44
High deprivation	0.96 (0.41–2.34)	0.96	0.50 (−0.16–1.55)	0.23
Medium deprivation	0.87 (0.36–2.12)	0.74	0.84 (0.28–2.55)	0.76
Low deprivation	0.53 (0.20–1.40)	0.20	0.25 (0.07–0.85)	0.03
Very low deprivation		1	1	
**Current support from mental health services**				
Yes	0.92 (0.53–1.58)	0.75	0.71 (0.34–1.48)	0.36
No	1		1	
**Diagnosis**				
Not recorded	2.44 (1.05–5.67)	0.04	3.48 (1.09–11.11)	0.04
Other SMI	3.00 (1.03–8.71)	0.04	2.44 (0.65–9.23)	0.19
Bipolar	2.28 (1.24–4.19)	0.01	2.97 (1.32–6.67)	0.09
Psychosis	1		1	
**Able to maintain a daily routine**				
No	3.63 (2.01–6.37)	0	2.27 (1.11–4.64)	0.03
Yes	1		1	
**Loneliness**	1.43 (1.24–1.64)	0	1.48 (1.24–1.76)	0
**Physical health**				
No decline	0.274 (0.16–0.48)	0	0.35 (0.17–0.70)	0.003
Decline	1		1	
**Professionally active**			Not included in model	
Yes	1.20 (0.69–2.07)	0.514		
No	1			
**Shielding**			Not included in model	
Yes	1.14 (0.59–2.19)	0.70		
No	1			

### Change in Loneliness

The ANCOVA analysis found no main effect of time on loneliness [*F*_(1,230)_ = 3.82, *p* = 0.05, η^2^ = 0.016] and no interaction between time and any of the other factors (all *p*s > 0.05). This suggests that loneliness did not change over time and this was not moderated by any of the examined factors.

## Discussion

This article explores how people with SMI's self- reported employment status, physical health, mental health, and well-being changed over the course of the pandemic. People were asked to complete questionnaires at two time points, the first asking them to compare their life during the first phase of the pandemic to prior to the pandemic and the second questionnaire asked them to compare their life during the second phase on the pandemic to their life in the first phase of the pandemic. Overall there was a mixed picture in terms of people's self-reported physical and mental health. Some people reported no change in their mental health at either time points, indicating that overall they did not believe their mental health had changed, whilst other reported that their mental health had worsened at both time points, indicating a continuous decline in mental health. Conversely others reported that their mental health had improved at both time points indicating an overall improvement in their mental health. It is difficult to draw any firm conclusions from this data but it is important to note that nearly a quarter of people (21.3%) reported a continuous decline in their mental health. General population surveys in the UK have identified a similar mixed picture (15). A similar picture was found in relation to change in physical health. Comparing data at individual time points, fewer people reported a decline in their mental health in the second phase of the pandemic compared to the first phase (34.1% compared to 43.4%). However, more people reported a decline in their physical health in the second phase of the pandemic compared to the first phase (38.2% compared to 32.9%). These differences were not tested for statistical significance, as we had not planned to test these in our pre-registered plan. The fact that more people reported a deterioration in physical health in the second survey is of concern as it may be that people have adopted unhealthy behaviors during the pandemic such being less physically active and more sedentary smoking more or eating a less healthy diet leading to a decline in physical health. A recent systematic review across all populations indicates a general decline in physical activity and increase in sedentary behavior (16). In addition a study exploring smoking and alcohol consumption in the general population in the UK found an increase in young people smoking between April and July 2020 compared to August 2019 to February 2020 and an increase in high risk drinking across all ages (17). Suggesting that changes in health risk behaviors may in part contribute to any decline in physical health. In addition it is possible that people have put off seeking help for physical health conditions, which again could lead to a decline in physical health. People with SMI already experience worse physical health than the general population so understanding if this decline is checked or indeed reversed with the reduction in restrictions is extremely important. If it is found that this decline in physical health is not reversed, or indeed is a continuing trend, strategies will need to be put in place to mitigate this. Furthermore, presenting to services later in the progression of physical health conditions could lead to a long-term increase in morbidity. Encouragingly the overall well-being score was similar at both time points, 22.73 (SD 8.67) at the first time point and 22.17 (SD 8.68) at the second time point, suggesting people's self-perceived well-being had not changed.

Factors associated with a change in mental health were explored to determine whether any protective factors could be identified which may then be used to develop recommendations to protect the mental health of people with SMI during a crisis. We found that not being able to maintain a daily routine was associated with a decline in mental health as was a self-perceived decline in physical health. Therefore, it is important that services provide appropriate support to people with SMI to help maintain a daily routine along with ensuring that their physical health needs are met.

Change in loneliness over time was explored and it was found that loneliness did not change over the course of the pandemic. Unfortunately we do not have data on loneliness prior to the pandemic so we cannot be certain whether people with SMI were more or less lonely during the pandemic than they were before the pandemic. However, loneliness in people with SMI is likely to be greater than in people without an SMI diagnosis (18) and as in the general population, loneliness is linked to being younger and living alone (19). It should be noted that the lack of changes in loneliness during the pandemic may be associated with the fact that loneliness does not correlate with the amount of time people with SMI spend with others (20). For these reasons it is important that levels of loneliness in people with SMI are monitored over the coming months to determine whether levels of loneliness remain static or improve, particularly given the impact loneliness can have on physical health (21).

Exploring change in professional activity between the two time points indicated that more people were professionally active in the second phase of the pandemic compared to the first phase of the pandemic. A *post-hoc* test showed that more people were professionally active prior to the pandemic than were professionally active during the first phase of the pandemic. This indicates that people were less likely to be professionally active during the first phase of the pandemic. It should be noted that professional activity is defined as being in any form of paid employment, being a voluntary worker or a student. This suggests that forms of professional activity that were lost in the first phase of the pandemic were regained in the second phase. The reason why this is the case is unclear. However, this is of interest given that throughout the duration of T2 there was a “stay at home” directive in place.

## Recommendations for Future Research

Further work is needed to explore whether protective factors relevant to a global crisis such as daily structure and needs met around maintaining physical health are more widely applicable to other local, community, family, or personal level crisis situations as this could be important information for service users interested in self-care as well as clinicians. For example, exploring whether introducing care packages to promote the establishment of a daily routine could provide a protective factor in terms of maintaining mental health.

In addition, further work is clearly needed to follow up this population and to observe patterns of recovery or further decline in terms of the mortality gap and physical health following the pandemic and map this to the general population. This work may also provide a window into factors driving the widening of the mortality gap (for example a decline in annual health checks) and suggest further research avenues for reducing and eventually eliminating this inequality.

Finally there is a need to understand the extent to which mental health services are aware of and monitor physical health to determine points at which protective factors could be promoted.

## Strengths and Limitations

The main strength of this study is that we provided participants with a range of options to take part and did not solely rely on an internet based study which is likely to have excluded those who are not digitally connected and thus miss out on an important sector of this population. Participants were able to take part by phone, online or by post. In addition we were able to contact and recruit people from a range of ages and demographics increasing the representativeness of this study. In addition we conducted extensive PPIE in the development of the questionnaires to ensure the topics that we included were relevant and important to people with SMI.

However, this study does have some limitations, despite making every effort to include some the most vulnerable people from the SMI population it is likely that some groups were not included due to the lack of access to some of the most vulnerable and excluded members of the SMI population, including those in assertive outreach services, possibly those with dual diagnosis, those in hospital long term and those who were too unwell to participate in our research for other reasons. This leads us to believe that perhaps the picture is not just one of division and inequality between the general population and those with SMI but also between those with SMI with the resources and capability to participate in research (even with support to do so) and those without.

As restrictions lift and the possibility of reaching these parts of the population (which is often done face to face and flexibly) increases, retrospective research which aims to hear these unsung voices of the pandemic is very much called for.

In addition this study used self-report rather than objective measures which mean that we cannot be certain that participants have accurately recalled how they felt prior to the pandemic or 6 months previously. Furthermore, previous studies have suggested that people with SMI may not accurately report their levels of functioning or quality of life (22, 23). However, other studies have found that people with SMI have accurately recalled their use of services (24, 25). The accuracy of reporting a limitation that is present in all studies of this nature.

Finally although we identified associations between a decline in mental health and physical health and not maintaining a daily routine this does not necessarily imply causality.

## Conclusion

No one single experience of people with SMI was identified, rather, this research demonstrates the complexity and diversity of experiences. However, it is of concern that some people reported a continuing decline in physical health and/or mental health, especially in a population already at risk from significant health inequalities. Therefore, to ensure that people with SMI do not experience a worsening of their physical and mental health as a result of the COVID-19 pandemic it is important that everyone is invited to, and encouraged to attend, an annual health check. Furthermore, raising awareness amongst mental health professionals of the importance of maintaining a daily routine and checking that physical health needs are met will be imperative in ensuring that both the physical and mental health of people with SMI is effectively supported.

## Data Availability Statement

The raw data supporting the conclusions of this article will be made available by the authors, without undue reservation.

## Ethics Statement

The studies involving human participants were reviewed and approved by Health Research Authority North West – Liverpool Central Research Ethics Committee (REC reference 20/NW/0276). Written informed consent for participation was not required for this study in accordance with the national legislation and the institutional requirements.

## Author Contributions

EP, PS, PH, SC, EN, LW, and RW contributed to the design of the survey. PS, PH, SC, and LW administered the survey to participants and collected data. EP, PS, and PH cleaned and organized the dataset. EP and PS conducted the statistical analysis. EP wrote the manuscript. GJ provided guidance from a lived experience perspective. SG provided senior academic guidance. All authors contributed to the conception and design of the study, the interpretation of the findings, manuscript revision, read and approved the submitted version.

## Funding

This study was supported by the Medical Research Council (grant reference MR/V028529) and links with the Closing the Gap cohort, which was part-funded by the Wellcome Trust (reference 204829) through the Center for Future Health at the University of York, UK Research and Innovation (reference ES/S004459/1), and the NIHR Yorkshire and Humber Applied Research Collaboration.

## Author Disclaimer

Any views expressed here are those of the project investigators and do not necessarily represent the views of the Medical Research Council, Wellcome Trust, UK Research and Innovation, National Institute for Health Research or the Department of Health and Social Care.

## Conflict of Interest

The authors declare that the research was conducted in the absence of any commercial or financial relationships that could be construed as a potential conflict of interest.

## Publisher's Note

All claims expressed in this article are solely those of the authors and do not necessarily represent those of their affiliated organizations, or those of the publisher, the editors and the reviewers. Any product that may be evaluated in this article, or claim that may be made by its manufacturer, is not guaranteed or endorsed by the publisher.
