# Supplementary material for: A Year Into the Pandemic: The Diversity of Experience Amongst People With Severe Mental Ill Health
Source: Front Psychiatry. 2022 Jan 27;12:794585. doi: 10.3389/fpsyt.2021.794585 (PMC8830291; doi:10.3389/fpsyt.2021.794585)
Supplement: Supplementary file 1 [file Table_1.DOCX]

Supplementary Table: Professional activity pre-COIVD-19 and at T1

|  | Professional activity Pre-COVID | |
| --- | --- | --- |
| Professional activity T1 | Active | Inactive |
| Active | 89 (72.4%) | 4 (1.7%) |
| Inactive | 34 (27.6%) | 235 (98.3%) |
